# Using prodigiosin against some gram-positive and gram-negative bacteria and *Trypanosoma cruzi*

**DOI:** 10.1590/1678-9199-JVATITD-2019-0001

**Published:** 2019-06-03

**Authors:** Rocío Herráez, Anna Mur, Alexandra Merlos, Miguel Viñas, Teresa Vinuesa

**Affiliations:** 1 Department of Pathology and Experimental Therapeutics, Medical School and IDIBELL, Campus Bellvitge, University of Barcelona, L’Hospitalet de Llobregat, Barcelona, Spain

**Keywords:** Trypanosoma cruzi, Chagas disease, Prodigiosin, Atomic force microscopy

## Abstract

**Background:**

This work aimed to explore the action of natural prodigiosin on both bacterial organisms and *Trypanosoma cruzi* cells.

**Methods:**

Natural prodigiosin pigment was extracted and purified from cultures of *Serratia marcescens*. Two media, peanut broth and peptone glycerol broth, both recommended in the literature for prodigiosin production, were compared. The prodigiosin obtained was employed to explore its antimicrobial properties against both bacteria and *Trypanosoma cruzi* cells.

**Results:**

Peanut broth yielded four times more prodigiosin. The prodigiosin showed remarkable activity (minimal inhibitory concentrations in the range of 2-8 µM for bacteria and half maximal inhibitory concentration of 0.6 µM for *Trypanosoma cruzi*). In fact, the prodigiosin concentration required to inhibit parasite growth was as low as 0.25 mg/l versus 4.9 mg/l of benznidazole required. Furthermore, atomic force microscopy revealed marked morphological alterations in treated epimastigote forms, although no pore-formation activity was detected in protein-free environments.

**Conclusions:**

This work demonstrates the potential usefulness of prodigiosin against some gram-positive and gram-negative bacteria and *Trypanosoma cruzi* although further studies must be done in order to assess its value as a candidate molecule.

## Background

Prodigiosin is a linear tripyrrol produced as a secondary metabolite by different bacteria, including *Serratia marcescens*, *Vibrio* spp., *Pseudomonas* spp. and *Streptomyces* spp., among others [1]. It is a highly stable pigment, and a member of the alkaloid family called prodigionines, presenting a blood-red color at acidic pH values. Its synthesis is closely related to amino-acid metabolism [2,3]. Although its biological role in the producer microorganisms has not yet been elucidated, it has demonstrated a high electron transport capacity. Prodigiosin is capable of uncoupling H^+^/Cl^−^ transporters, since it can bind to and transport chloride; subsequently, it modulates the pH of the bacterial cell [4]. Notwithstanding the scarce knowledge of its mechanism of action, prodigiosin appears as a pluripotent molecule, with various health-related properties; the most important being: an anticancer agent [5,6], an immunosuppressant, an antiprotozoal and an antibacterial agent, while also offering protection against UV [7,8,9]. Different studies have shown that prodigiosin inhibits the growth of a broad spectrum of gram-positive (*Staphylococcus* spp., *Bacillus* spp., etc.) as well as gram-negative (*Escherichia coli*, *Salmonella enterica*, etc.) bacteria [10]. Its capacity to inhibit the growth of eukaryotic microorganisms such as fungi (*Candida albicans*) and protozoa (*Plasmodium* spp. and *Trypanosoma* spp.) has also been reported [11,12,13].

The first report of the *in vitro* effect of prodigiosin on *Trypanosoma cruzi* dates from more than 65 years ago [14] when concentrations as low as 3 µM showed the capacity to completely inhibit the parasite. Azambuja *et al*. [15] reported interactions between *Trypanosoma cruzi* and a producer of prodigiosin, namely *Serratia marcescens*. Moreover, those authors succeeded in demonstrating that the loss of trypanosomal activity of *Serratia marcescens* DB11 was concomitant with the loss of capacity to produce prodigiosin, thereby indicating that the tripyrrolic pigment is a significant contributor to anti-trypanosomal activity. Furthermore, the eventual usefulness of prodigiosin in the treatment of Chagas disease has been explored. Genes *et al*. [16] indicated that the activity of prodigiosin may mediate an apoptotic phenomenon in *Trypanosoma cruzi* by induction of mitochondrial dysfunctions. Finally, prodigiosin and another bacterial pigment (violacein from *Chromobacterium violaceum*), as well as their combinations with silver and gold nanoparticles, have been studied for their *in vitro* inhibition of the growth of *Plasmodium falciparum* and *Trypanosoma brucei* [17,18]. Prodigiosin was found to be more effective than violacein at inhibiting both of these parasites; moreover, combinations of prodigiosin and metal nanoparticles resulted in increased parasiticidal activity, while toxicity for mammalian cells remained stable.

Nevertheless, the antimicrobial properties of prodigiosin have often been questioned, particularly because of the high concentrations required for it to be effective, as these exceed the levels causing toxicity in mammalian cells. For this reason, it has been studied in greater depth for its use in anticancer and immunosuppressive therapy, than as an agent to fight infectious agents [19,20].

The interest in prodigiosin as a drug is clearly demonstrated by the number of reports in the literature from the different fields concerned, as well as by the explorations of its complexation and encapsulation, for both drug-delivery and to enhance its activity [21].

Our group has acquired considerable expertise in dealing with not only the physiology and properties of prodigiosin but also its production by *Serratia marcescens*. We are specifically interested in the antimicrobial activity of prodigiosin, including its antitrypanosomal activity [6,15,16].

In the present study, we focused our efforts on purifying crude extracts of bacterial origin and determining their activity against the parasite, in the hope of lowering the concentration needed to inhibit growth of the parasite, as well as decreasing its toxicity to mammalian cells.

We compared two methods of prodigiosin production by evaluating the effect of peanut broth and peptone/glycerol broth on production of the pigment [22]. We also considered extraction methods by using organic solvents and setting up a purification system using an X-5 resin-affinity chromatography column [23] to purify the molecule in order to test whether such a procedure can increase its effectiveness at reducing toxicity.

Therefore, the present work aimed to explore and compare the action of the prodigiosin obtained on both bacterial organisms and *Trypanosoma cruzi* cells. In the case of the parasite, prodigiosin action was studied by determination of its anti-epimastigote activity, as well as by visualization of injuries and measurements of cell damage by atomic force microscopy (AFM). Furthermore, we have investigated the eventual alterations induced in membranes by the pigment through electrophysiological measurements of the conductance capacity in black lipid bilayers and by searching for channel forming activity.

## Methods

### Bacterial strains and cultures

The following bacterial strains were used to measure susceptibility: *Staphylococcus aureus* ATCC 29213, and ATCC 700698, *Enterococcus faecalis* ATCC 29212, *Escherichia coli* ATCC 25922, *Pseudomonas aeruginosa* ATCC 27853, plus one isolate of *Bacillus subtilis* and one of *B. pumilus,* from our own collection. They were thawed and maintained by daily passage in Columbia Blood Agar until the experiments were performed. *Serratia marcescens* 2170 was used as the prodigiosin producer.

### Parasite culture

For **Trypanosoma cruzi** CL-B5 maintenance, epimastigotes were cultured axenically at 28°C in liver infusion-tryptose medium (LIT; DifcoTM BD, MD, USA; Conda Pronadisa, Madrid, Spain) supplemented with 10% heat-inactivated fetal bovine serum (FBS; Gibco, Life Technologies, AU, NZ) plus antibiotics (100 μg streptomycin sulfate/ml and 100 U penicillin G sodium salt/ml, SP; Sigma Aldrich, CA, USA). The CL-B5 strain was maintained in exponential growth by weekly passages [24,25].

### Prodigiosin preparation

For prodigiosin production, *Serratia marcescens* 2170 was cultured overnight in peptone-glycerol medium under orbital shaking (250 rpm) at 30^o^C. Then, 75 ml of bacterial culture was spread onto peptone-glycerol agar and incubated for 48 hours at 28^o^C. After incubation, the bacteria were harvested by scraping the plate surfaces, suspended in methanol:HCl (24:1 v/v), and centrifuged (8,000 x g for 15 min) to eliminate debris. The pigment was then collected and concentrated in a rotary evaporator. These prodigiosin preparations were denominated PG 1.

In addition, after overnight culture in peptone-glycerol medium, *Serratia marcescens* 2170 was transferred to 1 l Erlenmeyer flasks (2.5 ml/l) and incubated for 72 h at 28^o^C in 250 ml of peanut medium at 250 rpm. The peanut medium consisted of 2% crushed peanut seeds in distilled water, autoclaved at 121^o^C for 20 min. The bacteria were centrifuged (8,000 x g for 15 min), washed twice and finally the pigment was extracted using a mixture of methanol:HCl (24:1 v/v), and shaken overnight at 250 rpm. After centrifugation to eliminate debris, the pigment was collected and concentrated in a rotary evaporator. These prodigiosin preparations were called PG 2.

In another batch of production, the supernatants from both of the previous media were collected and purified by the technique described by Wang *et al*. [23], with some modifications, as we did not use raw material but rather previously extracted prodigiosin.

After the X-5 resin had been hydrated (in MilliQ water), the column was packed and washed successively with water to remove impurities. A layer of sand was added above the packed column, and then the pigment solution was poured into the column. Elution was carried out with methanol and colored fractions were collected.

Purified prodigiosin produced from the peptone-glycerol medium was denominated PG 3; and the purified prodigiosin produced from the peanut medium was called PG 4.

The purified prodigiosin was evaluated using: spectrophotometric analysis with absorbance determined in the range 450-700 nm; thin-layer chromatography (TLC); and electrospray ionization mass spectrometry ESI-MS. The prodigiosin concentration was calculated using the Beer-Lambert law, considering a value for the molar extinction coefficient of 110,000 M^-1^·cm^-1^ and using the maximum 535 nm absorbance value.

### Antibacterial effect

Values of minimal inhibitory concentration (MIC) were determined by the broth microdilution method and interpreted according to the EUCAST guidelines. Briefly, an isolate of each bacterial strain was grown at 37 °C and 250 rpm *overnight* in 20 mL of Tryptic Soy Broth medium. Next, 800 µL of the culture was utilized to inoculate 20 m L of Mueller-Hinton medium. After 2h at 37°C and 250 rpm, bacterial inocula were adjusted with Ringer up to an optical density at 625 nm from 0.08 to 0.1 (0.5 McFarland; 10^7^-10^8^ ufc/m L). Five (5) µL of each bacterial culture was added to a 96-well microplate previously filled with serial dilutions of prodigiosin. The plates were incubated at 37°C for 24 h. The MIC was determined macroscopically, by assessing turbidity with the aid of a mirrored viewbox, scoring the lowest concentration well without turbidity. Growth and control wells were also included. Each concentration was run in triplicate and the experiments were repeated three times independently.

### Epimastigote susceptibility

Seven-day-old culture epimastigotes (2.5 x 10^5^ /ml) were incubated in 96-well microplates (WWR Int LLC) with serial dilutions of natural prodigiosins for 72 hours at 28°C. Growth and control wells with only the parasite and the reference drug benznidazole (ELEA, Buenos Aires, Argentina), respectively, were also included in the assay. Each concentration was run in triplicate and the experiments were repeated three times independently. Parasite growth was checked by cell counting in a Neubauer chamber.

The efficacy of the compounds was calculated by determining the epimastigote percentage growth, as follows:

[(CD3t - CD0) / (CD3c - CD0)] × 100

where CD3t is the epimastigote concentration of treated parasites at day 3; CD0 is the epimastigote concentration at day 0; and CD3c is the epimastigote concentration of untreated parasites at day 3 [26].

The IC_50_ values (half maximal inhibitory concentration) were calculated using GraphPad Prism software.

### Atomic Force Microscopy (AFM)

Since the activity of prodigiosin against microorganisms is attributed to plasma-membrane damage [27], we explored the effect of prodigiosin on epimastigote forms of *Trypanosoma cruzi* using AFM, by comparison between treated and untreated parasites. These alterations were also compared with those induced by the benznidazole treatments.

Epimastigote forms of *T. cruzi* (CLB strain) cultivated in LIT medium, after exposure to prodigiosin 1.26 µM and benznidazole 48 µM for 72 h, were visualized using an XE-70 atomic force microscope (Park Systems, Korea). We used these two concentrations [2 times 50% inhibitory concentration value (IC_50_)] since, although the parasite loses the capacity to grow, it is not completely destroyed and it is still feasible for visualizing organism surfaces and injuries. The parasites were collected by centrifugation, washed in phosphate buffered saline (PBS) 0.1 M, fixed with glutaraldehyde 2.5% in PBS 0.1 M for 1 hour, washed twice with 5 ml of MilliQ water and placed onto a MICA surface, allowed to dry at room temperature and then imaged in air using the AFM equipment. All images were collected in non-contact mode using pyramidal-shaped silicon cantilevers with a spring constant of ±40 N/m and a resonance frequency of ±300 kHz, with the upper side coated with aluminum to enhance the laser beam reflectivity. The data acquired during the surface scanning were converted into images of topography and amplitude, and analyzed using XEP and XEI software (Park Systems, Korea). In the topography images, it was possible to observe the shape, structure, and surface of individuals. Furthermore, amplitude images enabled visualization of fine surface details. The AFM images were acquired with a scan size of 20 μm^2^ at a scan rate of 0.3-0.6 Hz. AFM was also employed to measure the surface roughness of the treated and untreated individuals. The roughness average (Ra), defined as the average distance from the roughness profile to the center plane of the profile, was calculated from the topography images obtained in these experiments.

### Statistical analysis

Both height and surface nano-roughness averages of the parasite cells after treatment with prodigiosin and benznidazole were compared and statistically analyzed by one-way ANOVA and Tukey’s test. Differences were considered significant when p < 0.05.

### Lipid bilayer experiments

The eventual channel-forming activity was explored via preparation of artificial lipid bilayers and the subsequent addition of prodigiosin. The black lipid bilayer experiments were carried out via the method reported by Benz *et al.* [28]. Membranes were formed by forming a 0.8 mm^2^ hole in a divider separating two compartments of the Teflon chamber containing 5 mL each of a bathing solution of salt, with 1% (w/v) lipid diphytanoyl phosphatidylcholine solution in n-decane (Avanti Polar Lipids). All salts (analytical grade) were obtained from Sigma Aldrich, Spain. Voltages were applied across the membrane formed via Ag/AgCl electrodes connected by a salt bridge, and the resultant current was boosted by a factor of 10^9^-10^10^ by a current amplifier, and recorded on a Rikadenki strip chart recorder. The temperature was maintained at 20^o^C throughout.

In order to evaluate channel-forming activity, increasing volumes of PG 3, starting at 1 μl and arriving at a final volume of 50 μl, were added to the aqueous phase of both sides of the bilayer, until a final concentration of 0.44 μM was reached on each side.

For PG 4, after the addition of increasing volumes, starting at 1 μl and arriving at a final volume of 50 μl, we achieved a final concentration of 0.94 μM on each side.

## Results

### Prodigiosin production

**Figure 1.**
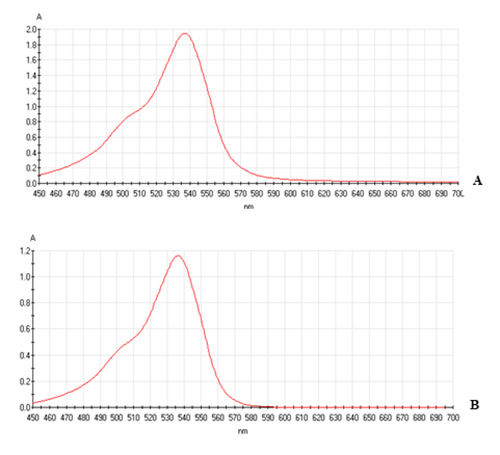
Visible adsorption spectra (range: 450-700 nm) of purified prodigiosin obtained from peptone-glycerol medium **(A)** and peanut broth **(B)**.

Both methods employed to produce prodigiosin to perform the further experiments yielded apparently clean pigment solutions that were relatively pure and adequate for the experiments. However, the pigment yield when bacteria were cultured in peanut broth was much higher, as shown in Table 1. In all cases, purity was determined and proven to be acceptable, as can be concluded from Figure 1, which shows absorption spectra with the typical peak at 535 nm and a shoulder at 515 nm; while no other peaks or disturbances were observed. Mass spectrometry confirmed the results (data not shown).

**Table 1.** Preparation of prodigiosin for experimental determination of antitrypanosomal and antibacterial action.

Prodigiosin preparation code	Medium	Extraction	Purification	Final prodigiosin concentration (µM)
PG 1	Peptone-glycerol	Methanol: HCl (24:1)	ND	42
PG 2	Peanut	Methanol: HCl (24:1)	ND	160
PG 3	Peptone-glycerol	Methanol: HCl (24:1)	X-5 Resin	44
PG 4	Peanut	Methanol: HCl (24:1)	X-5 Resin	177

ND: not done

### Antibacterial effect

Purified prodigiosin preparations (PG 3 and PG 4) displayed remarkable antimicrobial activity, as can be concluded from the MIC values (Table 2) against all the bacterial strains tested, which represented the major groups of pathogenic microorganisms except *Mycobacterium*.

**Table 2.** MIC of prodigiosin against the different bacterial strains tested.

Bacterial strain	MIC (μM)
*S. aureus* ATCC 29213	4
*S. aureus* ATCC 700698	4
*B. subtilis*	2
*B. pumilus*	2
*E. faecalis* ATCC 29212	2
*E. coli* ATCC 25922	2
*P. aeruginosa* ATCC 27853	8

MIC: minimal inhibitory concentration

### Epimastigote susceptibility

**Figure 2.**
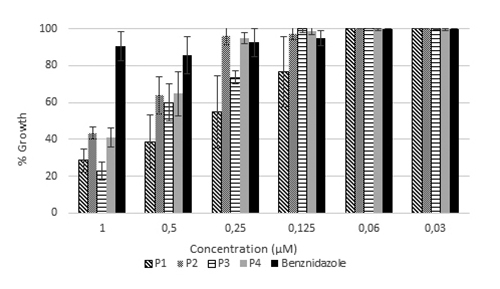
Biological activity of benznidazole and prodigiosin preparations against *T. cruzi* epimastigotes of the CL-B5 strain expressed as percentage of growth with corresponding standard deviation.

Similarly, the preparations were highly active against parasites in the epimastigote state (Fig. 2). Table 3 shows values of anti-epimastigote activity (IC_50_). Activity of both chromatographically purified prodigiosin preparations gave lower IC_50_ values than those in a native state (just extracted, without purification). The value for the drug currently utilized to treat Chagas disease (benznidazole) is also reported, to facilitate comparison.

**Table 3.** Biological activity of natural, extracted and purified prodigiosin preparations against *T. cruzi* epimastigotes (CL strain, clone B5).

Anti-epimastigote activity	IC_50_ (μM) ±SD
PG 1	0.80±0.25
PG 2	0.78±0.08
PG 3	0.60±0.1
PG 4	0.60±0.40
Benznidazole	18.9±7.06

IC_50_ 50% inhibitory concentration

### AFM

**Figure 3.**
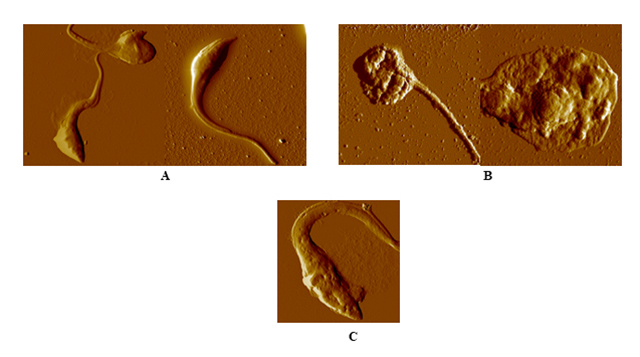
AFM amplitude images obtained at a 20 μm^2^ scan size, of: **(A)** untreated *T. cruzi* epimastigotes; **(B)** *T. cruzi* epimastigotes after 72 h of treatment with PG 3, 1.26 µM; **(C)** *T. cruzi* epimastigotes after 72 h of treatment with benznidazole, 48 µM.

**Figure 4.**
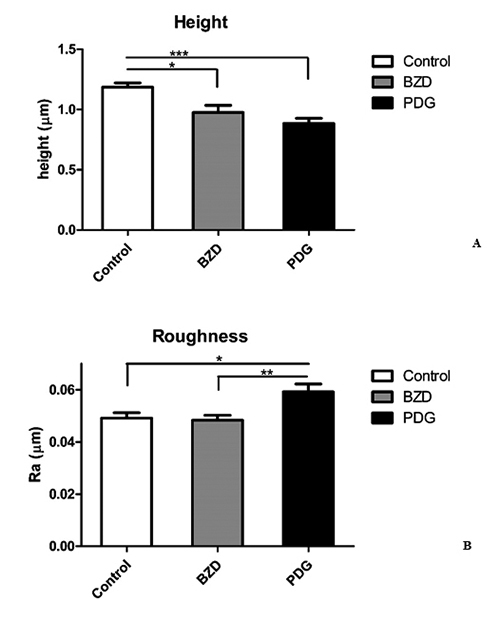
**(A)** Average height in micrometers, for the different formulations tested and surface scans. Bars represent the standard deviation of the mean of at least 30 measurements. *, P < 0.05; ***, P < 0.001. **(B)** Surface roughness (Ra) in micrometers, for the different formulations tested and surface scans. Bars represent the standard deviation of the mean of at least 30 measurements. *, P < 0.05; **, P < 0.01.

Epimastigote forms of *T. cruzi* treated with prodigiosin (PG 3) showed severe morphological alterations with loss of membrane integrity, as can be concluded by observing the corresponding images (Figure 3). Moreover, several parameters were affected after treatment, such as average height of the parasite cell (Figure 4a) and surface nano-roughness (Figure 4b).

### Lipid bilayer experiments 

When the extracts of prodigiosin (PG 3 and PG 4) were added to bathing solutions separated by black lipid bilayer membranes, no significant increases in conductance were observed. This indicates that, at least in lipid bilayers constructed in our laboratory, the incorporation of prodigiosin into the bilayer, when it happened, did not lead to formation of transmembrane channels at any of the concentrations assayed in our experiments. We conclude that there was no pore-forming activity of prodigiosin in protein-free environments.

## Discussion

Prodigiosin production was compared between the two media used (peptone-glycerol medium and peanut broth), revealing that peanut medium yields much more pigment (up to fourfold more) as shown in Table 1. Thus, when the goal is to produce natural prodigiosin, peanut broth would be efficacious. Our results confirm (and optimize) those reported by Giri *et al.* [22].

All four prodigiosin preparations obtained from S. *marcescens* 2170 cultures exhibited a marked antibacterial effect, similar to that already reported by other authors [27]. We obtained evidence that biological features of the prodigiosin obtained in our laboratory were maintained during the different extraction and purification procedures. When measuring antitrypanosomal action, there were no differences due to the media used in the production step (peptone-glycerol medium and peanut broth): the antitrypanosomal action was identical. Moreover, purification using X-5 resin increased antitrypanosomal effectiveness. This may be attributed to a higher purity level and the subsequent absence of interfering remains of bacteria and debris. In fact, when comparing the data, we can conclude that the benznidazole concentrations required to inhibit parasite growth are clearly higher than those needed with prodigiosin (4.9 mg/l and 0.25 mg/l, respectively).

When the capacity of prodigiosin to form channels was explored, our measurements did not indicate any insertion into the artificial membranes created. That is to say, no increases in conductance were detected. In principle, the addition of an ion transporter into a membrane should result in discrete increases of conductance; the lack of positive results may be a consequence of an incapacity of the molecule to become inserted into the bilayer. It has been shown that prodigiosin is a molecule that resides in bacterial membranes [29], while its antimicrobial action has been attributed to membrane injuries [27], which is apparently inconsistent with its failure to modify the membrane conductance observed herein. Nevertheless, artificial black lipid bilayers, despite acting electrophysiologically as cell membranes, are formed only by lipids and we cannot rule out the possibility that insertion of the prodigiosin, and its activity as an ion transporter, may require the cooperation of some membrane protein or some special domains of lipids other than the ones used in building artificial bilayers.

The effect of prodigiosin against gram-positive and gram-negative bacteria that we found is remarkable.

As to trypanocidal activity, the IC_50_ values of the purified prodigiosin preparations were more active than crude ones, probably due to the purity of their composition.

The action mechanism of prodigiosin as an antimicrobial agent is not fully established, although the acquired AFM images of treated epimastigotes showed remarkable changes on parasite cell surfaces as well as in height and roughness, suggesting a catastrophic scenario caused by the pigment. This is consistent and complements the results of Genes *et al.* [16] when reporting the activity of prodigiosin against *T. cruzi*, evidencing that the mode of action of prodigiosin involves the mitochondrial function. In fact, despite using different strains ( TcI and TCII) with different susceptibilities to Benznidazole, they found that classical inhibitors of respiratory function such as Rotenone, Thenoyltrifluoroacetone (TTFA), potassium cyanide (KCN) and antimycin A, produce an effect on mitochondrial membrane potential similar to that of prodigiosin, but they were much less parasiticidal. Thus, it may be plausible that prodigiosin action takes place not only on mitochondria, but also in other cell compartments and mainly on plasma membrane.

In conclusion, the findings of the present study strongly indicate that prodigiosin exerts a potent action by killing *T. cruzi* in its epimastigote form. These findings suggest that the bacterial secondary metabolite could become a resource in fighting Chagas disease. Therefore, additional studies need to be performed to verify its safety in relation to mammalian cells and intracellular and trypomastigote forms of the parasite.

### Abbreviations

 AFM: Atomic force microscopy; BZD: Benznidazole; ESI-MS: Electrospray ionization mass spectrometry; FBS: Foetal bovine serum; IC_50_: 50% inhibitory concentration; KCN: Potassium cyanide; LIT: Liver infusion-tryptose; MIC: Minimal inhibitory concentration; PBS: Phosphate buffered saline; PDG: Prodigiosin; PG 1: Prodigiosin 1; PG 2: Prodigiosin 2; PG 3: Prodigiosin 3; PG 4: Prodigiosin 4; Ra: Roughness average; TLC: Thin-layer chromatography; TTFA: Thenoyltrifluoroacetone.
